# An Assessment of Japanese Carbon Tax Reform Using the E3MG Econometric Model

**DOI:** 10.1100/2012/835917

**Published:** 2012-12-11

**Authors:** Soocheol Lee, Hector Pollitt, Kazuhiro Ueta

**Affiliations:** ^1^Faculty of Economics, Meijo University, 1-501 Shiogamaguchi Tenpak-ku, Nagoya 468-8502, Japan; ^2^International Modelling, Cambridge Econometrics, Covent Garden, Cambridge CB1 2HT, UK; ^3^Graduate School of Economics, Kyoto University, Yoshida-Honmachi Sakyo-ku, Kyoto 606-8501, Japan

## Abstract

This paper analyses the potential economic and environmental effects of carbon taxation in Japan using the E3MG model, a global macroeconometric model constructed by the University of Cambridge and Cambridge Econometrics. The paper approaches the issues by considering first the impacts of the carbon tax in Japan introduced in 2012 and then the measures necessary to reduce Japan's emissions in line with its Copenhagen pledge of −25% compared to 1990 levels. The results from the model suggest that FY2012 Tax Reform has only a small impact on emission levels and no significant impact on GDP and employment. The potential costs of reducing emissions to meet the 25% reduction target for 2020 are quite modest, but noticeable. GDP falls by around 1.2% compared to the baseline and employment by 0.4% compared to the baseline. But this could be offset, with some potential economic benefits, if revenues are recycled efficiently. This paper considers two revenue recycling scenarios. The most positive outcome is if revenues are used both to reduce income tax rates and to increase investment in energy efficiency. This paper shows there could be double dividend effects, if Carbon Tax Reform is properly designed.

## 1. Introduction

In recent years, the Japanese Government has proposed to introduce low-carbon policy instruments such as a carbon tax and ETS by introducing the bill of the Basic Act on Global Warming Countermeasures in 2009. The carbon tax plan was approved at the cabinet meeting in December 2011 (instead of ETS) due to strong ETS opposition from business circles. The bill has now been passed by the Japanese diet (March, 2012), making the carbon tax the first one to be introduced in Asia. The effects of carbon taxes on GHG emissions and the wider economy are now attracting the notice of many researchers and policy makers in this area.

The aim of this paper is to analyse the potential economic and environmental effects of implementing low-carbon policies, notably carbon taxation, in Japan. The analysis provides an input to the discussion by presenting a quantitative assessment of carbon taxation in Japan. The approach is model based and uses E3MG, a global macroeconometric model that links the world's economies to their energy systems and associated emissions. The assessment approaches the issue from two angles by considering first the impacts of the tax increases put forward in 2010 and then the measures that would be needed to reduce Japan's emissions in line with its Copenhagen pledge of −25% compared to 1990 levels.

This analysis builds on previous work by, among others, Park [1], Kawase et al. [2], and Takeda [3], who examined the effects of ETR using CGE (Computable General Equilibrium) models. E3MG shares many of the features of such models, for example, GTAP [4], the Monash model, and GEM-E3 [5], but relaxes some of the common assumptions to the CGE approach (such as fully rational behaviour), instead using an empirical approach (see Section 3). The structure of E3MG makes it more similar to the approach used in Sugawara [6], but E3MG is much larger in scale and with a much larger disaggregation of sectors.

The paper is structured as follows: Section 2 summarises the policy environment in Japan that provides the basis for this analysis; Section 3 describes the E3MG model, while Sections 4 and 5 describe the scenarios that were assessed and the results from these scenarios, respectively; Section 6 concludes.

## 2. Background: Japanese Climate Change ****Policy

### 2.1. A Brief Description of Japanese Climate Change Policy

Japan has been pushing for the adoption of measures to combat climate change since 1997, just after the agreement of Kyoto Protocol. The Law Concerning the Promotion of the Measures to Cope with Global Warming, for example, became effective in 1998. However, this law required business circles and householders to try to reduce GHG emissions without any imperative policy instruments like carbon taxes or ETS. Emission reduction efforts from business have depended on the Voluntary Action Plan led by Keidanren (Keidanren is the most influential general business association in Japan to which 1,281 leading companies and 127 industrial associations belong). Reductions from households have depended on government-led campaigns such as Cool Business, which requests people to control their office and room air conditioner over 28°C with light dress (e.g., no necktie and no jacket, etc.) during the summer season. 

In 2009, the Japanese Government submitted the bill of the Basic Act on Global Warming Countermeasures to parliament. The bill outlined a mid-term goal to reduce GHG emissions by 25% below the 1990 level in 2020 and a long-term goal of 80% below the 1990 level in 2050. Further goals include raising the share of renewable energy within total primary energy supply to 10% by 2020. Measures to achieve these targets have included proposals for carbon taxes and also an emissions trading scheme (ETS). The Committee on Institutional Design for Emissions Trading was established in 2000 by the Environmental Agency and began examining the introduction of an ETS at national level. The committee investigated an ETS as a domestic measure to achieve Japan's greenhouse gas reduction target under the Kyoto Protocol (see Committee on Institutional Design for Emissions Trading [7]).

In 2010, the Ministry of the Environment in Japan proposed an ETS that would be implemented in 2013. However, despite strong requests to implement an ETS from environmental NGOs and academia, the ruling party (i.e., the Democratic Party of Japan) proposed a postponement of its implementation on December 17, 2010, due to opposition from Keidanren and industry as well as potential negative economic impacts (advocates for the implementation of a cap-and-trade ETS include the Kiko Network [8], an environmental NGO, and Morotomi and Ayukawa [9]). At the end of 2010, the Japanese government opted to introduce a carbon tax in 2011 instead of an ETS. However, the implementation of this tax was put off until 2012 by the influence of the 2011 tsunami in Japan. 

Nevertheless, from the perspective of promoting global warming mitigation measures and pushing for energy conservation after the 2011 tsunami, there was a determined push in Japan to introduce a carbon tax (Special Provisions for Carbon Dioxide Tax of Global Warming Measures), and the Japanese FY2012 Tax Reform Revision passed the House of Councilors on March 30, 2012. It will be implemented from October 1, 2012. As a result, the government will increase the rates, depending on carbon content, of the present Petroleum and Coal Tax, which is imposed on all fossil fuels.

The first scenario in this paper simulates the potential effects of FY2012 Tax Reform. 

### 2.2. Carbon Tax Plan Details

The FY2012 Tax Reform (see http://www.mof.go.jp/english/tax_policy/tax_reform/fy2012/tax2012a.pdf) specifies that the Japanese Government will introduce a Carbon Dioxide Tax with the aim of controlling energy-originated CO_2_ emissions, which currently account for around 90% of GHG emissions. 

The government will add the following tax rates, corresponding to the amount of CO_2_ emissions, to existing fossil fuel prices (including existing taxes):

- crude oil, petroleum products: JPY 760/kl,
- gaseous hydrocarbons: JPY 780/t,
- coal: JPY 670/t.

The additional taxes are set to be introduced, in part, on October 1, 2012 and increased progressively such that they are fully implemented by 2016.  Table 1 specifies the interim measures in further detail. These figures are used to define the first scenario in this paper.

## 3. The E3MG Model

This section briefly describes the E3MG model that was used to carry out the analysis. For further information about the model, the reader is referred to Barker et al. [10] and the website http://www.e3mgmodel.com.

### 3.1. Basic Model Structure

The E3MG model (energy-environment-economy model at the global level) is a computer-based tool that has been constructed by international teams at the University of Cambridge and Cambridge Econometrics. The model is econometric in design and is capable of addressing issues that link developments and policies in the areas of energy, the environment, and the economy. The essential purpose of the model is to provide a framework for policy evaluation, particularly policies aimed at achieving sustainable energy use over the long term. However, the econometric specification that the model uses also allows for an assessment of short-term transition effects.

**Figure 1:**
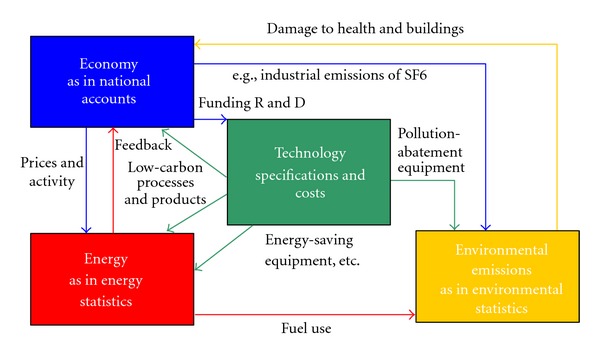
E3 interactions within E3MG.

The current version of E3MG consists of 22 world regions, although in this analysis we focus solely on Japan. The basic structure of E3MG is presented in Figure 1. The model integrates energy demand and emissions with the economy; fuel demand is determined by prices and economic activity with feedback through the energy supply sectors. Energy combustion results in greenhouse gas emissions.

The economic module in E3MG contains a full representation of the National Accounts, as formulated in Cambridge by Richard Stone, and formally presented in European Communities et al. [11]. A key feature of E3MG is its sectoral disaggregation, with 42 economic sectors, linked by input-output relationships; this aspect is particularly important in modelling carbon taxes as the different sectors use different fuels in varying degrees of intensity and have different technological options for changing consumption patterns.

Exogenous inputs to the model include population, government tax and spending rates, and international energy prices. The outputs include a range of economic and labour market indicators, defined at sectoral level, plus indicators for energy consumption and emissions.

**Figure 2:**
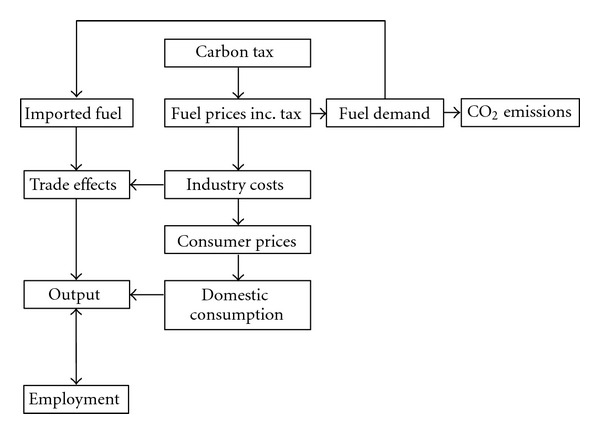
Potential effects of a carbon tax.

Figure 2 shows the mechanism through which a carbon tax could affect macroeconomic outcomes. The taxes are levied on consumption of fuel use, leading to reductions in fuel demand but also higher costs for industries and households. Higher industry costs may be absorbed as loss of profits or passed on to final consumers. Higher prices mean losses of real output for domestic consumers and for exporters.

**Figure 3:**
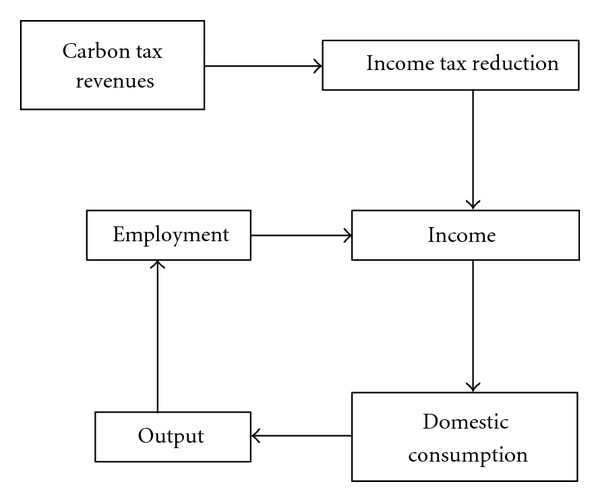
Potential effects of revenue recycling.

However, the revenues from carbon taxes may also be used to reduce other tax rates, with positive economic benefits. In the scenarios in this paper, a large share is used to reduce income taxes. The effects this has on the economy are shown in Figure 3; reduced income taxes lead to higher incomes, which are spent on consumer goods and lead to increases in domestic production, creation of jobs, and further income rises (i.e., a multiplier effect).

E3MG's treatment of energy demand is largely top-down in nature. Econometric equations are estimated for aggregate energy demand and demand for the four main fuel types (coal, fuel oil, natural gas, electricity). Energy demand, for 19 different user groups, is a function of economic activity, relative prices, and measures of technology. The model solves all equations simultaneously and adjusts the individual fuels to sum to the total for each user. Feedbacks to the economy are provided by adjusting input-output coefficients and household energy demand.

The following equations provide an example of E3MG's econometric, error-correction equations for aggregate energy consumption (*EnCon*) at time *t*. First a long-run equation is estimated based on levels of economic activity (*Act*), energy prices (*EnPrice*), investment (*Inv*), and *R*&*D*. The lagged errors from this equation (e) are then used in the short-run equation which uses differences of the same independent variables, plus the lagged dependent variable.

*Long Run*:

(1) EnCont=a1+(b1∗Actt)+(b2∗EnPricet)+(b3∗Invt)+(b4∗R&Dt)+et.

*Short Run*:

(2) ΔEnCont=a2+(b1∗ΔActt)+(b2∗ΔEnPricet)+(b3∗ΔInvt)+(b4∗ΔR&Dt)+(b5∗et−1)+(b6∗ΔEnCont−1)+∈t.

The exception to this top-down treatment is in power generation, as the historical data do not provide the basis to estimate econometric equations in new technologies. In this sector E3MG includes a bottom-up representation with 28 specific generation technologies, made up of both conventional and renewable supplies. The model bases future investments on the relative prices of each technology, including the effects of carbon taxation. This part of the model is described in Barker et al. [12]. 

Emissions are estimated using a fixed coefficient to fuel demand. Nonenergy emissions are included in the model so that global totals are met but are treated as exogenous in this paper.

E3MG also includes endogenous measures of sectoral technological progress. The indices used in the model are functions of accumulated capital, enhanced by R&D, adapted from Lee et al. [13]. Endogenous technological progress is allowed to influence several of the model's equation sets, including energy demand, international trade, price formation, and the labour market.

### 3.2. Data Sources and Equation Estimation

As an econometric model with sectoral detail, E3MG requires extensive data inputs. A large time-series database covering 1970–2008 annually (with more recent aggregate figures where available) has been constructed, in the main based on international datasets. For Japan the main data source for economic data is the OECD Structural Analysis database, with other macro-level indicators being obtained from the IMF and the World Bank. If there are gaps in the data these are filled using national figures. The main cross-sectional data (the input-output table and bilateral trade flows) are sourced from the OECD.

The main source for energy data is the IEA. CO_2_ emissions have also been made consistent with IEA figures. 

E3MG consists of 22 estimated sets of equations (each disaggregated by sector and by country). These cover the components of GDP, prices, the labour market, and energy demand. 

The estimation method utilises developments in time-series econometrics, in which dynamic relationships are specified in terms of error correction models (ECM) that allow dynamic convergence to a long-term outcome. 

The specific functional form of the equations is based on the econometric techniques of cointegration and error-correction, particularly as promoted by Engle and Granger [14] and Hendry et al. [15]. In brief, the process involves two stages. The first-stage is a levels relationship, whereby an attempt is made to identify the existence of a cointegrating relationship between the chosen variables, selected on the basis of economic theory and a priori reasoning. For example, for employment demand the list of variables contains real output, real wage costs, hours worked, energy prices, and a measure of technological progress. If a cointegrating relationship exists, then the second stage regression is known as the error-correction representation and involves a dynamic, first-difference, regression of all the variables from the first stage, along with the lagged difference in the dependent variable, and the error-correction term (the lagged residual from the first stage regression). 

### 3.3. Previous Analysis with E3MG

The E3MG model has been under development for much of the past decade. It is now used for policy analysis at European level, including the 2010 European Commission communication on the impacts of moving to a 30% GHG target. The model has also been used repeatedly for assessing decarbonisation pathways at different international levels [10] and in the UK [16]. Most recently E3MG was applied in Barker et al. [17] to provide an economic assessment of the IEA's 450 ppm scenario [18].

Also of potential application to these scenarios and their underlying policy context is the model's assessment of rebound effects [23]. In this paper the E3MG model was used to show that long-run rebound effects can cancel out up to 50% of the environmental gains from efficiency measures; the analysis goes on to recommend carbon pricing as a means to reduce the rebound effect.

### 3.4. Comparison to CGE Modelling

In terms of basic structure, purpose, and coverage, there are many similarities between E3MG and comparable CGE models, such as GTAP [4], the Monash model, and GEM-E3 [5]. Each is a computer-based economic model that considers energy-environment-economy interactions at the global level, broken down into sectors and world regions. In addition the regional and sectoral disaggregations are broadly similar. Both modelling approaches are based on a consistent national accounting framework and make use of similar national accounts data.

However, beneath the surface there are substantial differences in modelling approach and it is important for the reader to be aware of this when interpreting results. The two types of model come from distinct economic backgrounds. While the models are quite consistent in their accounting, identity balances, they differ substantially in their treatment of unobservable behavioural relationships. The CGE model favours setting these in line with economic theory, for example, by assuming that individuals act rationally in their own self-interest. In contrast, the econometric model interrogates historical datasets to try to determine these factors on an empirical basis.

Both approaches have their relative strengths and weaknesses; for example, the assumption of optimising rational behaviour in CGE models has been increasingly questioned since the recession, while econometric models are reliant on having high-quality time-series data. Although subtle, these differences in theoretical approach can lead to different conclusions being drawn from the model results; for example, the econometric model does not assume optimal behaviour in the baseline, implying that negative-cost emission reductions are available. Jansen and Klaassen [24] and Bosetti et al. [25] describe some of the differences in the context of ETR, including revenue recycling options.

This distinction is important when comparing the analysis in this paper to previous model-based assessments in Japan, which have almost exclusively used a CGE approach, as discussed in Section 1. In Europe it is now common for CGE and macroeconometric models to be run in tandem so that results are not dependent on a single set of modelling assumptions (e.g., [19]).

## 4. Scenarios

### 4.1. Baseline

The baseline that has been used for this analysis has been scaled to be consistent with the current policies scenario in *World Energy Outlook*, 2010 ([18], henceforth referred to as WEO). E3MG's equation results are set to match WEO figures for energy demand and emissions, but also to use the same figures for economic drivers in order to retain consistency throughout the model's internal relationships. In summary, by 2020,

- total energy-related CO_2_ emissions are expected to fall to 998 mtCO_2_, from 1,147 mtCO_2_ in 2008 and 1,063 in 1990;
- total primary energy demand is expected to be stable at current levels;
- final consumption of oil is expected to slowly decrease, but consumption of electricity to increase;
- additional electricity will be generated from nuclear power.

The 2010 edition of WEO does not include the latest data regarding the recession or the impacts of the Fukushima nuclear accident (which clearly raises questions on the suitability of the final bullet point) but, while this may impact on the magnitude of results, in our view it does not change the direction of results, or the overall conclusions from this paper. Nevertheless, one of our recommendations is to repeat this exercise once there is more certainty about the global economy and Japanese energy and climate policy.

### 4.2. Policy Scenarios

We consider four policy scenarios. The first scenario assesses the economic and environmental impacts of the tax increases that were announced for FY2012. The other scenarios consider the measures that would be necessary to reduce GHG emissions by 25% from 1990 levels, in line with Japan's Copenhagen pledge. There are three variants of this scenario, one with the carbon tax levied on its own and two options with different methods of revenue recycling (where revenues from carbon taxes are used to reduce revenues from other taxes). In scenario 2b, some of the revenues are used to fund a public investment programme in energy efficiency (the investment in energy efficiency is assumed to lead to reductions in energy demand using the ratios of investment and energy savings for OECD countries published in IEA [18]) in buildings, while in S2c they are used to reduce labour costs.

The scenarios are summarised in Table 2, while Table 3 illustrates the tax inputs for each scenario. Inputs for S2a, S2b, and S2c are not derived from Japanese policy but are estimated by E3MG based on achieving the CO_2_ emissions reduction target of 25% in 2020 compared to 1990.

## 5. Results

### 5.1. Environmental Impacts

**Figure 4:**
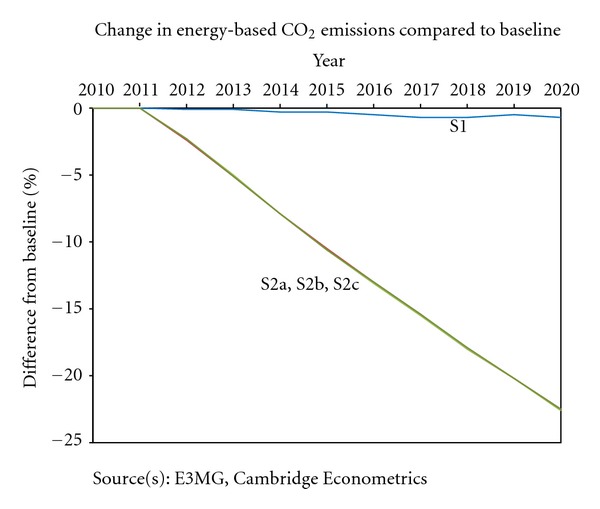
Energy-based CO_2_ emissions, Japan.

Figure 4 shows the impact on energy-related CO_2_ emissions, compared to baseline, in each of the scenarios.

The first result is that the FY2012 measures that are modelled in S1 have only a small impact on emissions levels; this is because their impact on fuel prices (i.e., the relative effect once the taxes are added to fuel costs and existing taxes) is very small, in the range of 1–3%. This provides little incentive for behavioural change, and there are only small reductions in fuel consumption. In the other scenarios, emissions fall by slightly more than 20% compared to baseline (which is 25% below 1990 levels). However, quite a high carbon price is required to do this in such a short period of time; the model results suggest that electricity prices would need to increase by up to 50% and motor fuel prices increase by around 40%.

### 5.2. Macroeconomic Impacts

Our results suggest that the average annual revenues raised during 2012–2020 would be approximately ¥310 bn ($4 bn) in S1 and ¥11,500 bn ($150 bn) per year in the variants of S2. The latter equates to around 3% of GDP and is clearly enough to have an impact on macroeconomic indicators.

**Figure 5:**
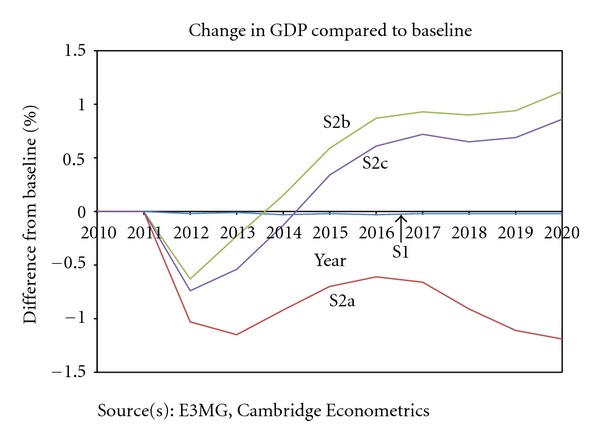
Japan GDP.

Previous model-based studies, including Andersen and Ekins [26] and Ekins and Speck [27], have found that it is possible to reduce CO_2_ emissions while simultaneously increasing GDP through carefully designed ETR. The results in this paper are consistent with this finding, on the condition that the revenues generated by carbon taxes are recycled effectively. As Figure 5 shows, modest GDP gains of up to 1.2% in 2020 (compared to baseline) are possible under these scenarios. It is also notable that the potential costs to GDP in S2a are also quite small.

The reason that GDP increases in the scenarios with revenue recycling is that the positive effects of reducing income taxes outweigh the negative effects of higher energy costs. The recycled revenues (including the share allocated to investment or reducing social security contributions) sum to the revenues from the carbon taxes, so the same amount of demand is in the system. However, there is a reallocation within the system that produces economic benefits for two main reasons.

- As Japan imports such a large share of its energy, any measures that reduce energy consumption are likely to boost the trade balance and hence GDP.
- Much of the higher energy costs fall on business which may not pass these costs on to final consumers (e.g., due to international competition); there is therefore a redistribution from companies (lower profits) to workers (higher incomes), but workers have a lower savings ratio meaning there is additional spending overall.

The second of these effects is dependent on cost pass-through rates which are largely determined by econometric estimates. Our results suggest that pass-through rates in Japan are often low, which contributes to the GDP benefits. (The reasons for this are not clear but could be linked to the period of deflation. To test the impact of this we carried out a sensitivity analysis with 100% pass-through, as is common in CGE models. The results showed that GDP still increased in scenarios 2b and 2c but by less than the amount reported in this paper.) Table 4 shows the impacts on the price level and other macroeconomic indicators.

Another reason that the measures with revenue recycling have a positive impact on GDP is the fact that Japan is relatively less exposed to international trade. This means that the competitiveness effects of carbon taxes (higher domestic costs leading to higher imports and lower exports) are less, while the impacts of the revenue recycling are higher (consumers spend less of the extra income on imports). This means that, despite a worsening trade balance, increases in household expenditure lead to quite positive results to GDP overall.

**Figure 6:**
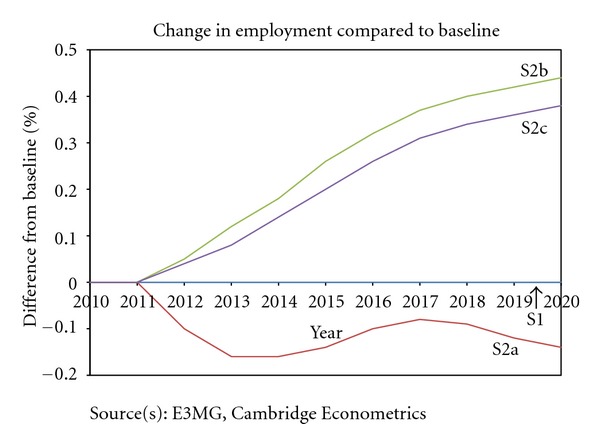
Japan employment.

Impacts on investment are quite small, except when a share of the revenues is used to fund investment programmes. The impacts on employment are smaller in scale than the impacts on GDP (see Figure 6) but generally follow the same pattern. In S2c additional jobs are created as a result of a share of the revenues being used to reduce labour costs. 

Additionally, the model results enable us to compare the macroeconomic effects between different approaches to revenue recycling. The revenue recycling scenarios both cause GDP and employment to increase in comparison to the scenario without revenue recycling (S2a). S2b, which considers using revenues to both reduce income tax and increase investment in energy efficiency, has slightly greater positive effects than S2c, which also uses revenues to reduce income tax but combined with reducing employers' social security contributions.

Our model results are consistent with the “double dividend hypothesis.” (This is explained in Goulder [28].) Apart from increasing welfare due to lower pollution externalities (a green dividend), environmental taxes raise revenue that can be used to lower other pre-existing tax distortions, resulting in economic welfare gains from a smaller deadweight loss of the tax system, or “efficiency” dividend.

### 5.3. Sectoral Impacts

As with any new policy there will be winners and losers created.  Table 5 summarises the main sectoral impacts from the model results in terms of changes in real output. It is important to note that the level of sectoral detail in the model is limited by the available data and that there will be subsectors and specific firms that will be affected by more than the sectoral figures shown here.

The patterns in outcomes are as would be expected from such a modelling exercise. The energy sectors suffer a loss in demand from their output, although much of this is met by lower imports. Electricity sees the greatest reduction in output in all of the scenarios. Other sectors that stand to lose out are those that are intensive users of energy and are exposed to international competition.

The sectors that stand to benefit are typically those that are not carbon intensive and supply products to consumers (see Table 5). Within S2a, some consumer sectors, such as hotels and catering, benefit from a shift in spending away from energy, but the overall outlook is negative. As household incomes increase in S2b and S2c, the same sectors benefit from additional spending that is directed towards them. In addition, the investment sectors benefit in S2b as a result of the spending programme. Output in textiles and clothing is quite sensitive, with strong negative effects in S2a but notable positive effects in S2b and S2c. This suggests a high income elasticity of demand in the sector. 

**Figure 7:**
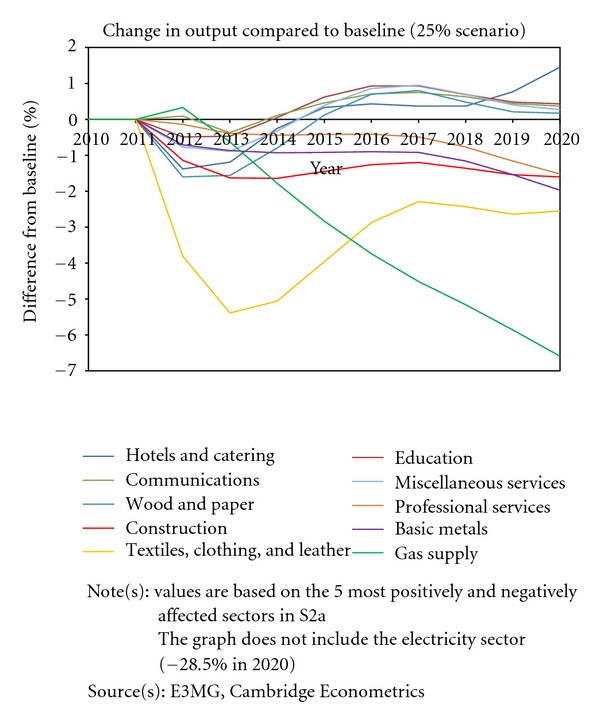
Selected output effects under 25% reduction target.

The patterns for sectoral employment are similar to the ones for sectoral output.  Figure 7 also illustrates a more detailed time-series representation of the most positively and negatively affected sectors in S2a.

### 5.4. Comparison to Previous Results in Japan

While the modelling results are consistent to similar exercises carried out in Europe using a similar modelling approach (e.g., [26]), they should also be compared to previous analysis carried out in Japan.

In Japan, the CGE model has been the main tool to analyse the economic and environmental effects of carbon taxes. Using a CGE model, Park [1] shows double dividend effects of a carbon tax for a 20% emission reduction target in 2010, using the revenues to cut employers' social contributions (as has been done in S2c). This carbon tax scenario resulted in a GDP increase between 0.16 and 0.49% in 2010 compared to baseline. Also based on CGE modelling, Park [29] introduces a 30,000 yen/tCO_2_ carbon tax to achieve the Japanese Kyoto Protocol target of 6% GHG reductions in 2010 compared to 1990. The results show double dividend effects for a carbon tax with revenue recycling. 

Kawase et al. [2] analysed the effects of a 3,000 yen/tCO_2_ carbon tax using CGE and industrial I-O analysis. The results showed a reduction in CO_2_ between 0.11 and 0.27% and an increase in GDP between 0.01 and 0.09% compared to baseline, with the revenues recycled through cuts in consumption or income taxes.

Sugawara [6] suggested that Japanese GDP will increase by 1-2% annually without introducing low-carbon policy. But, based on analysis using a macroeconometric model, this would be reduced to zero (and the unemployment rate would increase by 3 percentage points) if Japan introduced a carbon tax to meet its 25% GHG 2020 target. However, if the tax revenues are recycled to public investment, the negative impacts would be lessened.

For the main part, the studies above show results that are consistent with the analysis presented in this paper. A carbon tax with revenue recycling could have a positive impact on GDP and seems very likely to lead to reduced CO_2_ emissions. But the results for achieving a 25% reduction target in this paper suggest a larger economic effect than previous studies. This partly reflects the structure of the E3MG model, with many of the assumptions that are common to CGE models being relaxed. However, it also reflects the ambitious nature of the 25% reduction target, high international energy prices (meaning that reductions in imported fuels have greater economic benefit), and the spare economic capacity that is available in the Japanese and global economies following the financial and economic crisis.

## 6. Conclusions

This paper analyses the effects of carbon taxes on GHG emissions and the wider economy for the Japanese FY2012 Tax Reform Revision. It also includes three scenarios of carbon taxes that are simulated to reduce GHG emissions by 25% in 2020 compared to 1990 levels. The analysis uses the E3MG model developed by the University of Cambridge and Cambridge Econometrics.

The results from the model suggest that the FY2012 Tax Reform Revision has only a small impact on emission levels and no significant impact on GDP and employment.

The potential costs of reducing emissions by a large percentage (to meet the Copenhagen target for 2020) are quite modest, but noticeable (GDP falls by around 1.2% compared to baseline and employment by 0.4% compared to baseline). But this could be offset, possibly with some economic benefits, if revenues are recycled efficiently. The results suggest that there could be double dividend effects, if the revenues from carbon taxes are recycled efficiently. 

## Figures and Tables

**Table 1:** FY2012 proposed tax rates.

	Crude petroleum/petroleum products (per kilo liter)	Gaseous hydrocarbon (per ton)	Coal (per ton)
Present	JPY 2,040	JPY 1,080	JPY 700
October 1, 2012	JPY 2,290	JPY 1,340	JPY 920
April 1, 2014	JPY 2,540	JPY 1,600	JPY 1,140
April 1, 2016	JPY 2,800	JPY 1,860	JPY 1,370

**Table 2:** Summary of scenarios.

	Carbon tax rates	Revenue recycling
S1	FY2012 reform	None
S2a	To reach 25% GHG reduction	None
S2b	To reach 25% GHG reduction	95% of revenues used to reduce income taxes, 5% used for investment in energy efficiency
S2c	To reach 25% GHG reduction	75% of revenues used to reduce income taxes, 25% used to reduce employers' social security contributions

**Table 3:** E3MG carbon tax inputs (JPY/toe).

	S1			S2a	S2b	S2c
	Oil	Gas	Coal	All fuel types		
2012	325	305	371	17,721	20,529	19,812
2013	325	305	371	26,982	25,843	29,316
2014	651	610	743	29,508	29,215	32,487
2015	651	610	743	30,709	28,652	32,468
2016	989	915	1,131	32,500	27,460	33,318
2017	989	915	1,131	35,257	28,750	35,868
2018	989	915	1,131	39,749	33,140	41,531
2019	989	915	1,131	43,192	36,528	45,426
2020	989	915	1,131	44,240	35,615	45,811

Notes: Figures based on 1 USD = 76.8 JPY.

S1 values derived from FY2012 tax rates.

S2 values required to achieve 25% reduction in CO_2_ emissions between 1990 and 2020.

Source(s): E3MG, Cambridge Econometrics.

**Table 4:** Macroeconomic impacts, Japan.

	S1	S2a	S2b	S2c
GDP	0.0	−1.2	1.1	0.9
Employment	0.0	−0.1	0.4	0.4
H'hold consumption	0.0	−1.6	2.0	1.7
Investment	0.0	−0.6	0.9	0.7
Exports	0.0	−0.5	−0.4	−0.5
Imports	0.0	−0.3	1.1	1.1
Price level	0.1	2.5	1.4	2.0

Values are % difference from the Baseline.

Source(s): E3MG, Cambridge Econometrics.

**Table 5:** Real output effects, selected sectors.

S1		S2a		S2b and S2c	(S2b)	(S2c)
Gas supply	−0.3	Basic metals	−2.0	Printing and publishing	6.1	5.6
Electricity	−1.0	Textiles and clothing	−2.6	Hotels and catering	5.3	5.1
		Gas supply	−6.6	Food/drink and tobacco	5.3	4.8
		Electricity	−28.5	Textiles and clothing	4.8	4.1

Notes: values are percent of difference from the baseline.

Source(s): E3MG, Cambridge Econometrics.
